# Genistein Prevents Apoptosis and Oxidative Stress Induced by Methylglyoxal in Endothelial Cells

**DOI:** 10.3390/molecules29081712

**Published:** 2024-04-10

**Authors:** Maria Liccardo, Luigi Sapio, Shana Perrella, Ivana Sirangelo, Clara Iannuzzi

**Affiliations:** Department of Precision Medicine, Università degli Studi della Campania “Luigi Vanvitelli”, Via L. De Crecchio 7, 80138 Naples, Italy; maria.liccardo@unicampania.it (M.L.); luigi.sapio@unicampania.it (L.S.); shana.perrella@unicampania.it (S.P.); clara.iannuzzi@unicampania.it (C.I.)

**Keywords:** methylglyoxal, oxidative stress, antioxidant activity, genistein, caspase-3, p38

## Abstract

Glycolytic overload promotes accumulation of the highly reactive dicarbonyl compounds, resulting in harmful conditions called dicarbonyl stress. Methylglyoxal (MG) is a highly reactive dicarbonyl species and its accumulation plays a crucial pathophysiological role in diabetes and its vascular complications. MG cytotoxicity is mediated by reactive oxygen species (ROS) generation, a key event underlying the intracellular signaling pathways leading to inflammation and apoptosis. The identification of compounds able to inhibit ROS signaling pathways and counteract the MG-induced toxicity is a crucial step for developing new therapeutic strategies in the treatment of diabetic vascular complications. In this study, the effect of genistein, a natural soybean isoflavone, has been evaluated on MG-induced cytotoxicity in human endothelial cells. Our results show that genistein is able to counteract the MG-induced apoptosis by restraining ROS production, thus inhibiting the MAPK signaling pathways and caspase-3 activation. These findings identify a beneficial role for genistein, providing new insights for its potential clinical applications in preserving endothelial function in diabetic vascular complications.

## 1. Introduction

Methylglyoxal (MG) is a highly reactive dicarbonyl species spontaneously produced by the fragmentation of glycolytic intermediates, mainly glyceraldehyde 3-phosphate and dihydroxyacetone phosphate, formed during glucose metabolism and to a lesser degree from intermediates of lipid and protein metabolism [1,2]. Moreover, MG is a major intermediate of the non-enzymatic glycation reaction leading to the formation of the advanced glycation end-products (AGEs), which are associated with several aging-related diseases including diabetes, cancer, and neurodegenerative diseases [2,3,4]. For this reason, MG formation and its accumulation play a crucial pathophysiological role in these pathologies, especially in diabetes and its vascular complication, both microvascular (retinopathy, neuropathy, and nephropathy) and macrovascular (ischemic heart disease, cerebrovascular disease, and peripheral vascular diseases) [5,6,7,8,9]. Endothelial cell (EC) injury and its dysfunctions have been related to the pathogenesis of vascular complications in diabetes mellitus [10]. Oxidative stress in EC is considered the starting event that triggers the intracellular signaling transduction pathways that lead to inflammation and apoptosis [11,12].

In diabetic patients, due to their hyperglycemic state, plasma levels of MG and AGEs are strongly increased and accumulate abnormally in multiple tissues and organs, playing a prominent role in the pathogenesis of diabetes and correlated vascular complications [13,14]. The molecular mechanism by which physiopathological levels of MG induces endothelial dysfunction is not fully understood. However, several studies have evidenced that the MG cytotoxicity is mainly induced through mitochondrial membrane potential impairment and reactive oxygen species (ROS) generation, the key event underlying the intracellular signaling pathways leading to inflammation and apoptosis [15,16]. Specifically, the MG-induced oxidative stress causes endothelial cell apoptosis through the activation of the ROS-mediated MAPK (JNK, p38, and ERK1/2) signaling pathways and the activation of mitochondrial caspase-3 [17,18].

Significant efforts have been made to identify compounds able to inhibit ROS signaling pathways in order to counteract the MG-induced toxicity in EC with the aim of developing new potential therapeutic strategies in the treatment of diabetic vascular complications [18,19,20,21]. In this respect, plant polyphenols have attracted considerable interest as food supplements to ameliorate and prevent diabetic-related complications due to their multiple biological activities, including antioxidant, anti-inflammatory, and antidiabetic power [22,23,24,25].

**Figure 1:**
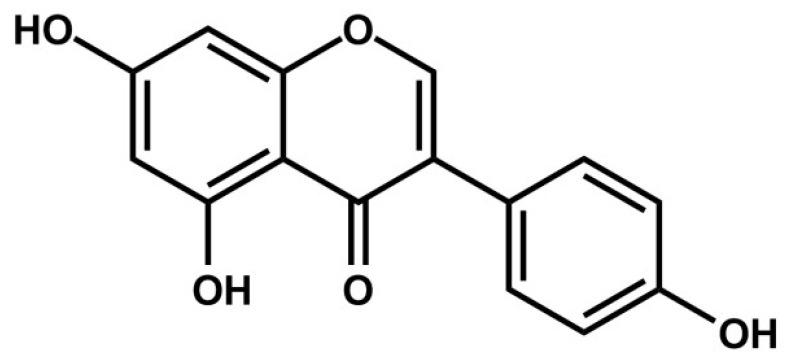
Chemical structure of genistein.

Among these natural polyphenols, genistein is a soybean isoflavone and naturally occurring tyrosine kinase inhibitor, known for several biological and therapeutic properties due to its antioxidant power and ant-inflammatory capacity [26,27,28,29] (Figure 1). Genistein exhibits estrogen effects, neuroprotective action against ischemic injury, antilipogenic and hypolipidemic properties, as well as chemoprevention in cancer treatment [28,30,31]. Moreover, genistein is suitable in playing a protective role in diabetes and its vascular complication as result of its antioxidant, anti-inflammatory and hypoglycemic activity [32,33,34,35,36,37]. Genistein also plays a protective role in MG-induced immune dysfunction in diabetic patients, reducing the oxidative stress and DNA damage as well as ROS generation and apoptosis in mononuclear cells [38]. Plant extracts containing genistein also showed a protective effect against MG-induced glucotoxicity in EC by reducing ROS formation and apoptosis [39]. Moreover, it has been reported that genistein shows anti-glycation activity by trapping MG both in vitro and in vivo, thus inhibiting the formation of AGEs [40,41].

In light of these considerations, in this study, we have analyzed the effect of genistein on MG-induced apoptosis in a human endothelial cell model. Our results show that genistein is able to counteract the MG-induced cytotoxicity by restraining ROS production, thus inhibiting the ROS-mediated MAPK signaling pathways and the activation of caspase-3. These findings identify a beneficial role for genistein, providing a promising basis for further in vivo and pre-clinical studies with the aim of evaluating its possible use in diabetic vascular complications.

## 2. Results

### 2.1. Genistein Markedly Reduces the MG-Induced Toxicity in EA.HY926 Cells

**Figure 2:**
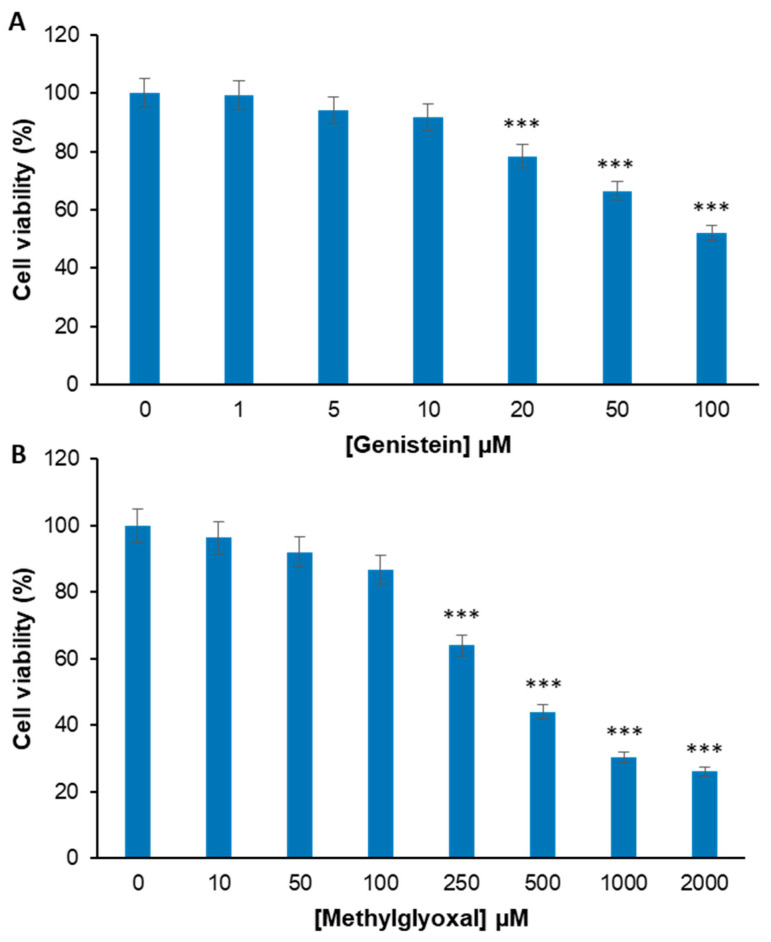
Evaluation of genistein and MG cytotoxicity in endothelial cells. Cell viability has been evaluated by MTT assay in EA.HY926 cells exposed for 24 h to increasing concentrations of genistein (0–100 μM) (**A**) and MG (0–2000 µM) (**B**). Data are expressed as average percentage of cell viability reduction ± SD relative to untreated cells from triplicate wells from 5 separate experiments. Other experimental details are described in the Materials and Methods section. *** *p* ˂ 0.05 versus untreated cells.

Before testing the effect of the combination treatment of genistein plus MG in endothelial cells, the toxicity of both genistein and MG has been evaluated in EA.HY926 cells, a representative endothelial cell line, in order to optimize the experimental conditions. Specifically, EA.HY926 cells have been exposed to different concentrations of genistein (0–100 μM) and MG (0–2 mM) for 24 h and the cell viability has been assessed by the MTT assay (Figure 2). For genistein, after 24 h of treatment, significant cytotoxicity was observed only in the presence of 20, 50, and 100 µM (Figure 2A), IC50 = 100 µM, whereas, for MG, the toxicity was observed in the range of 250–2000 µM (IC50 = 400 µM). In particular, 250 µM MG promotes a 36% reduction in cell viability, while higher concentrations (500–2000 µM) reduce the cell viability by over 50% (Figure 2B).

Based on the above results, the working concentrations for our study were set at 0–20 µM for genistein and 250 µM for MG in order to minimize the cell death after 24 h of treatment.

**Figure 3:**
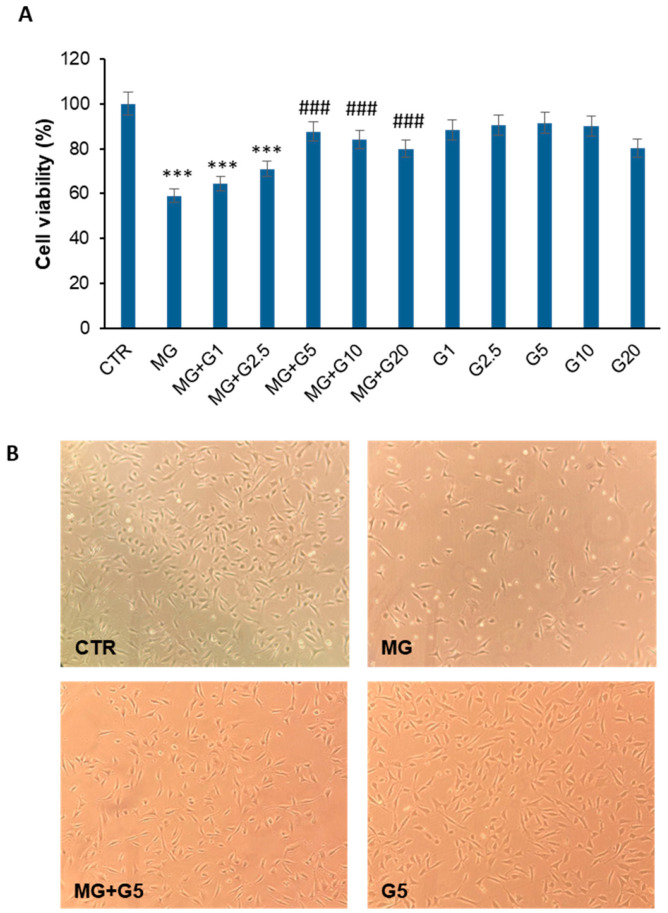
Effect of genistein on MG-induced cytotoxicity. (**A**) Cell viability evaluated by MTT assay in EA.HY926 cells exposed for 24 h to 250 µM MG (MG), pretreated for 2 h with 1 (MG + G1), 2.5 (MG + G2.5), 5 (MG + G5), 10 (MG + G10), and 20 (MG + G20) µM genistein. Data are expressed as average percentage of cell viability reduction ± SD relative to untreated cells (CTR) from triplicate wells from 5 separate experiments. *** *p* ˂ 0.05 versus CTR, ^###^ *p* ˂ 0.05 versus MG. (**B**) Phase contrast microscopy images of EA.HY926 cells after 24 h of incubation. CTR: untreated cells; MG: cells exposed to 250 µM MG; MG + G5 cells pretreated with 5 µM genistein; G5: cells treated with 5 µM genistein. Other experimental details are described in the Materials and Methods section.

To investigate the effect of genistein on MG-induced cytotoxicity, EA.HY926 cells pretreated for 2 h with genistein at different concentrations (0, 1, 5, 10, and 20 µM) were incubated with 250 µM MG and the cell viability was monitored by MTT assay after 24 h of treatment (Figure 3A).

Our results show that, while cells exposed to MG showed a strong reduction (about 40%) in cell viability after 24 h of treatment, the absence of toxicity was observed for cells treated in the presence of genistein in the range of 5–20 µM (about 80% cell viability). In particular, while the lower concentration of genistein (1 µM) only slightly affects the MG cytotoxicity, 5, 10, and 20 µM genistein are able to protect endothelial cells by MG toxicity. In order to evaluate modifications in cell morphology and cell number upon different treatments, cell samples were also analyzed by phase-contrast microscopy (Figure 3B). Similarly to the MTT assay, phase-contrast microscopy shows that cells exposed to MG for 24 h display both modifications in cell morphology and reductions in the cell number, whereas those pretreated with genistein exhibit no qualitative and quantitative alterations.

**Figure 4:**
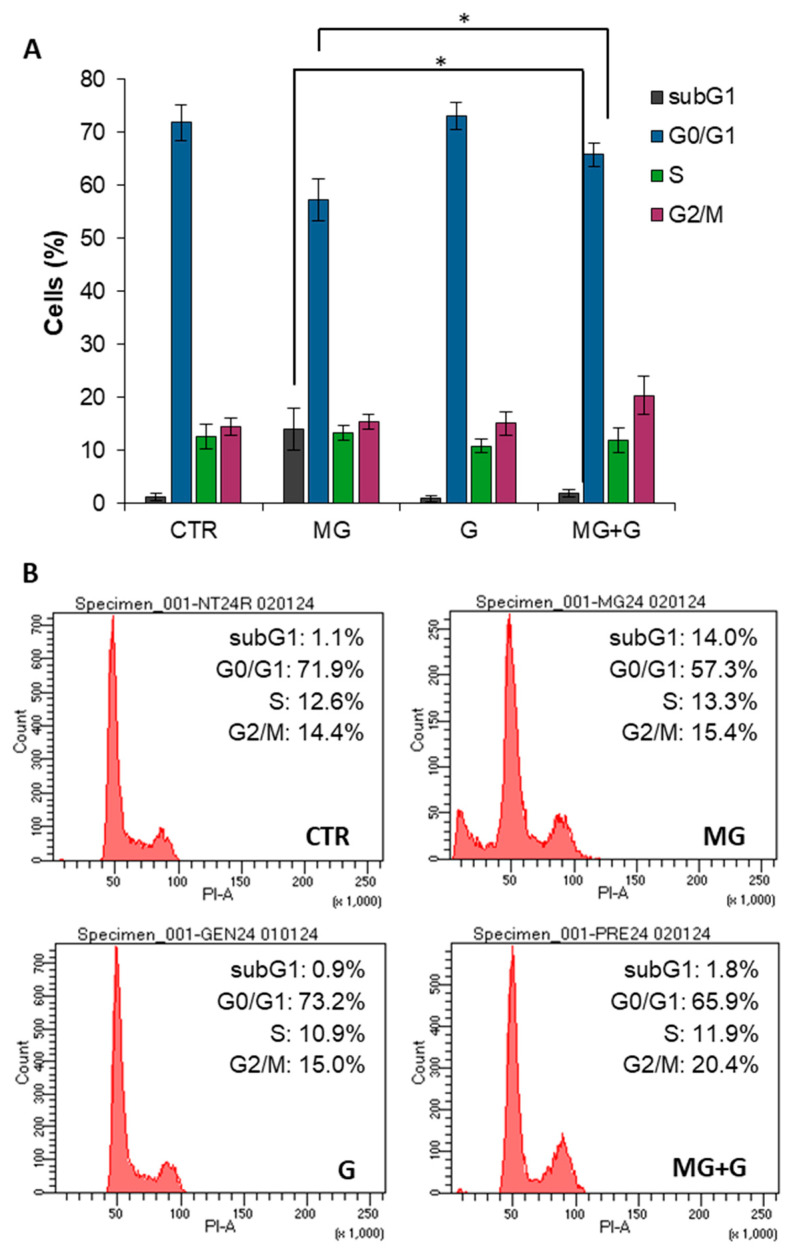
Evaluation of effects of genistein and MG, single and in combination, on cell cycle distribution. EA.HY926 cells were exposed for 24 h to 250 µM MG (MG) and underwent 2 h of pretreatment with 5 µM genistein (G), either alone or in combination (MG + G), for 24 h. Thereafter, the relative cell-cycle distribution was assessed using FACSCelestaTM employing PI as a DNA dye. (**A**) Quantitative analysis of multiple experiments. (**B**) Representative experiment. * *p* < 0.05.

The protective effect of genistein on the MG-induced cytotoxicity has been further analyzed through the evaluation of the cell cycle distribution (Figure 4). Flow cytometric analysis (FACS) indicated that EA.HY926 cells treated with MG for 24 h showed a significant G0/G1 reduction (−14.6%) and a concomitant subG1 appearance (+13%) compared to untreated cells. Interestingly, pretreatment with genistein strongly mitigated the cell cycle alterations observed with MG. In particular, cells pretreated with genistein exhibited a higher percentage of G0/G1 (57.3% vs. 65.8%) and no subG1 occurrence compared to cells exposed to MG only. These data further confirm the cell protection observed for genistein by MTT assay and clearly suggest that genistein 5 µM strongly reduces the MG-induced toxicity in EA.HY926 endothelial cells.

### 2.2. Genistein Prevents MG-Induced ROS Production and Nrf2 Activation in EA.HY926 Cells

**Figure 5:**
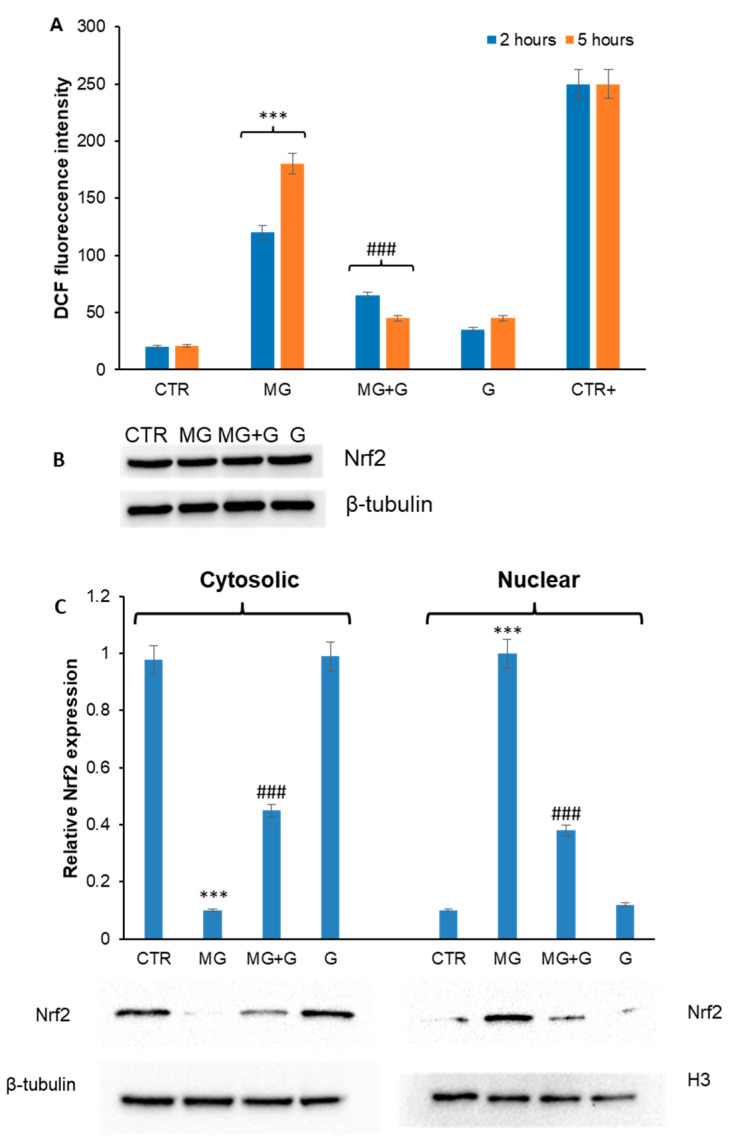
Role of genistein in MG-induced oxidative stress. EA.HY926 cells were exposed to MG (MG) and pretreated with genistein (MG + G), and it has been evaluated ROS production by DCFH-DA assay (**A**), Nrf2 expression (**B**), and Nrf2 nucleus translocation (**C**) by western blot analysis. Panel B shows Nrf2 expression in the total cell extract, while panel C shows the Nrf2 level for cytosolic and nuclear fractions. CTR: untreated cells; G: cells treated with genistein; CTR+: cells treated with 1.0 mM H_2_O_2_. Data are expressed as average ± SD from five independent experiments carried out in triplicate. MG and genistein concentrations were 250 and 5 µM, respectively. Other experimental details are described in the Materials and Methods section. *** *p* ˂ 0.05 versus CTR, ^###^ *p* ˂ 0.05 versus MG.

Oxidative stress has been identified as the main mechanism by which MG promotes cytotoxicity in endothelial cells, due to the increase in ROS production and subsequent mitochondrial membrane depolarization, a key event underlying the molecular signaling pathways leading to inflammation and apoptosis [42,43,44]. Genistein is known to possess a strong antioxidant activity mainly acting as a free radical scavenger [45,46,47]. In this respect, with the aim of identifying the molecular basis of the genistein cellular protection in MG toxicity, we have evaluated the effect of genistein in MG-associated oxidative damage. At first, the ability of genistein to reduce the ROS production associated with MG treatment has been tested (Figure 5). In particular, as MG is known to induce ROS production in endothelial cells [13,17,18], the intracellular ROS levels have been measured in EA.HY926 cells pretreated with 5 µM genistein and then treated with MG for 2 and 5 h, by the DCFH-DA fluorescence assay (Figure 5A). Our results show that treatment with MG 250 µM promotes an increase in the DCF fluorescence after both 2 and 5 h of incubation, indicative of ROS production. By contrast, in the sample pre-incubated for 2 h with genistein, the ROS levels were similar to those of untreated cells, thus suggesting that genistein is able to counteract the MG-induced ROS production in EA.HY926 cells.

Considering the protective effect of genistein in the MG-induced ROS production, we have evaluated the expression of Nrf2 in EA.HY926 cells exposed to MG in the absence and in the presence of genistein (Figure 5B,C). Nrf2 is a transcriptional regulator that modulates oxidative stress response and is known to be sequestered in the cytosol bound to the inhibitory protein Keap1. In oxidative stress conditions, Nrf2 dissociates from Keap1 and moves into the nucleus, thus inducing the expression of downstream antioxidant enzymes [48]. For this reason, Nrf2 expression has been evaluated in total cellular extracts as well as cytosolic and nuclear fractions. Analysis performed on the total extract shows that no variation in Nrf2 expression is observed in all experimental groups (Figure 5B). By contrast, the exposure of cells to MG resulted in Nrf2 translocation from cytosol to the nucleus, thus suggesting its activation. In cells pretreated with genistein, a significant reduction in Nrf2 translocation was observed (Figure 5C). The overall data suggest that the presence of genistein is able to impair the MG-induced ROS production in EA.HY926 cells.

### 2.3. Genistein Protects Endothelial Cells by the MG-Induced Apoptosis through the MAPK-Mediated Signaling Pathways

**Figure 6:**
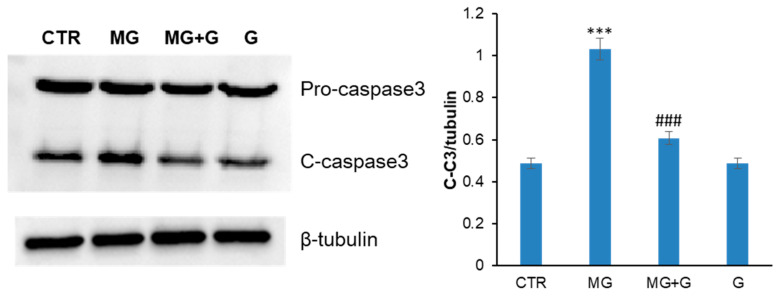
Effect of genistein on caspase 3 activation. EA.HY926 cells were exposed to MG (MG) and pretreated with genistein (MG + G) and caspase 3 activation has been evaluated by Western blot analysis. CTR: untreated cells; Gen: cells treated with genistein. Data are expressed as average ± SD from five independent experiments carried out in triplicate. MG and genistein concentrations were 250 and 5 µM, respectively. Other experimental details are described in the Materials and Methods section. *** *p* ˂ 0.05 versus CTR, ^###^ *p* ˂ 0.05 versus MG.

MG promotes apoptosis in endothelial cells mainly through the production of ROS able to promote oxidative stress-related pathways that underly the apoptotic cascade [28,48]. At the molecular level, MG is known to induce apoptosis in endothelial cells via mitochondrial dysfunction, ROS/MAPKs/Nf-kB signaling pathways, and ER stress [49]. In endothelial cells, the redox-sensitive family members of MAPKs triggered by MG are ERK, JNK, and p38 MAPKs [17,50]. Genistein is known to exert an antioxidant effect through the inhibition of JNK, ERK, p38 MAPKs, and Nf-kB activation [29,51,52]. Thus, to further investigate the genistein-mediated cell protection in response to MG treatment in endothelial cells, Western blotting analysis has been performed to identify putative effects of genistein on the MG-induced apoptosis. At first, the activation of caspase 3 was evaluated in EA.HY926 cells treated in the presence and absence of 5 µM genistein upon MG treatment for 24 h by measuring the cleaved active caspase 3 (C–C3) (Figure 6). As expected, while MG promotes caspase 3 cleavage, no activation was observed in cells pre-incubated with genistein, thus suggesting protection on MG-induced apoptosis in EA.HY926 cells.

**Figure 7:**
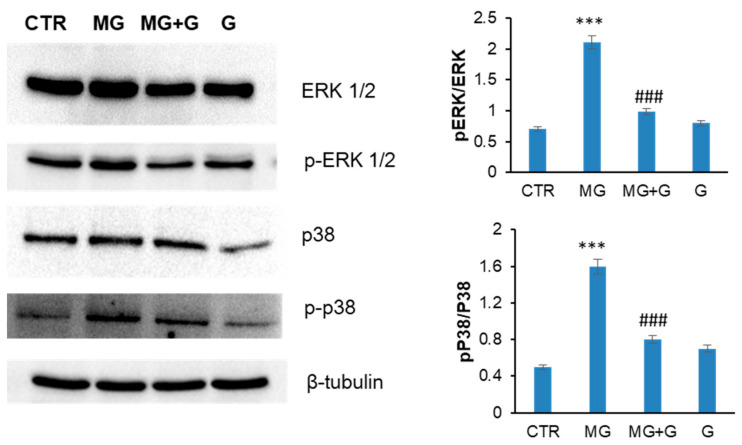
Effect of genistein MAPK activation. EA.HY926 cells were exposed to MG (MG) and pretreated with genistein (MG + G), and ERK1/2 activation and p38 MAPKs have been evaluated by Western blot analysis. CTR: untreated cells; G: cells treated with genistein. Data are expressed as average ± SD from five independent experiments carried out in triplicate. MG and genistein concentrations were 250 and 5 µM, respectively. Other experimental details are described in the Materials and Methods section. *** *p* ˂ 0.05 versus CTR, ^###^ *p* ˂ 0.05 versus MG.

To monitor whether the protection observed by genistein on MG-induced apoptosis occurred through the MAPK signaling pathways, the activation of ERK and p38 MAPKs was also evaluated in the same experimental groups (Figure 7). Western blot analysis suggests that MG promotes the activation of both ERK and p38 after 24 h of incubation in EA.HY926 cells, which is consistent with the observed caspase-3 activation as directly activated by p38 phosphorylation. By contrast, in cells pre-incubated with genistein, no activation of MAPKs is observed, thus suggesting that genistein is able to protect EA.HY926 cells by interfering with MAPK pro-apoptotic pathways.

## 3. Discussion

High intracellular glucose concentration promotes the formation of highly reactive dicarbonyl compounds, which accumulate and lead to harmful conditions called dicarbonyl stress [53]. Methylglyoxal is the most reactive dicarbonyl metabolite of glucose and, in diabetic conditions, is known to trigger non-enzymatic glycation, which eventually lead to the irreversible overproduction of AGEs [54]. The molecular mechanism by which MG induces ROS production and oxidative stress in endothelial cells is mediated by the activation of NADPH oxidase, which induce superoxide generation and mitochondrial dysfunction, which eventually lead to endothelial dysfunction, inflammatory responses, and cell death [15,16,55]. In the present study, we show that genistein, a natural isoflavone, significantly counteracts the MG-induced toxicity in EA.HY926 endothelial cells. Indeed, genistein is able to restore the cell viability and damage following MG exposure, as indicated by the reduction in cell toxicity, cell cycle alterations, and morphological modifications. In particular, genistein protects endothelial cells by MG-induced apoptosis by suppressing ROS generation and MAPK signaling pathways.

The ROS generation is an early event in the MG-related toxicity, leading to inflammation and cell death [15,16,56]. In diabetes and other age-related diseases, the increase in MG and ROS levels also promotes the glycation of several biomolecules like DNA and proteins, leading to the formation of AGEs, highly toxic species also able to induce RAGE expression. As both circulating MG and its derivative hydroimidazolone-1 can bind RAGE, thus promoting ROS production in human endothelial cells [57], the suppression of ROS formation by phytoantioxidants can be considered a crucial approach useful in preventing MG-induced endothelial apoptosis. Indeed, Pang and coworkers have reported that polydatin, a glucoside of resveratrol, protects human umbilical endothelial cells by MG-induced apoptosis through the inhibition of ROS formation and the maintenance of mitochondrial membrane potential [20]. Similarly, the flavone apigenin and unripe Carica papaya have been shown to inhibit AGE-induced oxidative stress and inflammatory signaling through ERK1/2 and Nf-kB [58,59]. In the present study, we show that genistein markedly reduces intracellular ROS formation, thus inhibiting the ROS-mediated MAPK signaling pathways and the activation of mitochondrial caspase-3 in EA.HY926 cells.

The process of ROS-induced cellular production can be considered the link between oxidative stress and apoptosis. High concentrations of MG promote apoptosis in endothelial cells through mitochondrial dysfunction, ROS/MAPK/Nf-kB signaling pathways, and ER stress [17,18,49]. The redox-sensitive family members of MAPKs triggered by MG in endothelial cells are mainly ERK, JNK, and p38 MAPKs [50]. We show that, while MG triggers cell apoptosis in endothelial cells by promoting the phosphorylation of ERK1/2 and p38, pretreatment with genistein decreases the MAPK phosphorylation and suppress apoptosis.

The ability of genistein to protect by MG-induced apoptosis can be ascribed to its strong antioxidant power. Genistein possesses natural antioxidant activity with different biological and pharmacological properties. Recently, many studies have been focused on the remedial roles of genistein for diabetes mellitus, renal, reproductive disorders, neurodegenerative dysfunctions, and malignancy [26,27,28,29]. As a strong antioxidative agent, genistein has been shown to inhibit oxidative stress and postpone the progression of diabetes-associated complication [32,33,34,35,36,37]. Several mechanisms have been proposed for MG in stimulating ROS production and promoting oxidative stress. In particular, NADPH oxidase has been suggested to be the major source of ROS generation [16,55,60]. Further mechanisms involve the production of hydrogen peroxide and superoxide anions during the reactions of protein glycation and the depletion of the glutathione content by the glyoxalase metabolic system [57,61,62]. Our results show that genistein inhibits ROS formation as indicated by the DCFH-DA assay, thus impairing defense mechanisms mediated by Nrf2. The ability of genistein to contrast ROS production could be ascribed both to its free radical scavenger activity and, also, to the increase in antioxidant enzyme expression. Further studies will be needed to better clarify the molecular mechanisms underlying the protective effect of genistein-MG-induced ROS production and oxidative stress.

**Figure 8:**
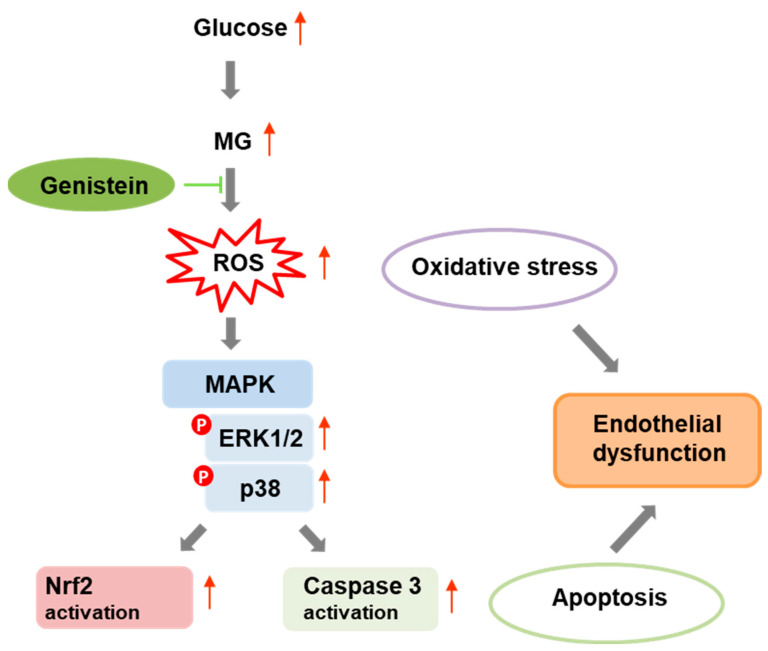
Schematic diagram for genistein protection in MG-induced endothelial cell apoptosis. Proposed mechanism for genistein in the MG-induced endothelial dysfunction. As depicted, genistein inhibits the production of ROS, thus preventing MAPKs and Nrf2 signaling pathways and protecting EA.HY926 endothelial cells by oxidative stress and apoptosis.

The overall data suggest that the beneficial effect of genistein in the MG-related apoptosis is mainly associated with its antioxidant activity. Indeed, the ability of genistein in maintaining redox homeostasis seems to be the key mechanism in preventing inflammation and apoptosis from dicarbonyl stress in endothelial cells. Indeed, intracellular ROS generation can induce ER stress and NADPH oxidase activation that eventually drive endothelial cells to apoptosis by Akt/MAPKs/Nf-kB pathways. In this study, genistein is shown to protect endothelial cells by disrupting the signaling cascades that activate MAPKs (Figure 8). In this respect, our study identifies a promising beneficial activity for genistein in counteracting the dicarbonyl-related endothelial toxicity.

## 4. Materials and Methods

### 4.1. Materials

Genistein (G6649), methylglyoxal (M0252), 3-(4,5-dimethylthiazol-2-yl)-2,5-diphenyl-tetrazolium bromide (MTT) (Sigma-Aldrich Co., St. Louis, MO, USA). Antibodies: anti-caspase-3 (#9662); anti-p44/42 MAPK (ERK1/2) (#9107), anti-phospho-p44/42 MAPK (ERK1/2) (Thr202/Tyr204) (#4377), anti-NRF2 (#12721), anti-p38 MAPK# (#9212), anti-β-tubulin (#2146) (Cell Signaling Technology, Boston, MA, USA). Anti-Histone H3 (#06-755) (Merk Millipore, Burlington, MA, USA). Anti-phospho-p38 MAPK (Thr180/Tyr182) (Sigma-Aldrich Co., St. Louis, MO, USA). Secondary antibodies: anti-mouse (#7076) and anti-rabbit (#7074) (Cell Signaling Technology, Boston, MA, USA). All other chemicals were of analytical grade. Methylglyoxal was further purified by distillation under low pressure and its concentration was determined spectrophotometrically using the molar extinction coefficient at 284 nm: 12.3 M^−^^1^ cm^−^^1^.

### 4.2. Cell Cultures and Treatments

EA.HY926 human endothelial cells (CRL-2922, ATCC Virginia, Manassas, VA, USA) were cultured using Dulbecco’s Minimum Essential Medium (DMEM) containing 10% (*v*/*v*) fetal bovine serum, 100 U/mL penicillin, 100 mg/mL streptomycin, and 2.0 mM glutamine. Cell cultures were maintained at 37 °C in a humidified atmosphere containing 5.0% CO_2_. Treatments with genistein were performed starting from a stock solution of 20 mM in DMSO diluted in cell media at 100 µM. The solution of genistein 100 µM in cell media was further diluted for different cell treatments. Treatments with MG were performed starting from a stock solution of 5 M in water that was diluted in cell media at 50 mM. The solution of MG 50 mM was further diluted for different cell treatments. For genistein–methylglyoxal experiments, cells were pretreated for 2 h with genistein before incubation with methylglyoxal.

### 4.3. MTT Assay

MTT assay was used to determine cellular metabolic activity as an indicator of cell viability through the ability of cells to reduce the tetrazolium salt (3-[4,5-dimethylthiazol-2-yl]-2,5-diphenyltetrazolium bromide, MTT) to blue formazan crystals. A stock MTT solution (5 mg/mL in phosphate-buffered solution, PBS) was prepared and filtered through a 0.22 µ Millipore^®^ filter. After treatments, the culture medium of each cell culture was removed, and the culture was washed with PBS. The MTT stock solution was diluted ten times in cell medium (without red phenol) and incubated for 3 h at 37 °C. Then, the medium was removed, and the cells were treated with isopropyl alcohol and 0.1 M HCl for 20 min to dissolve the formazan crystals. Levels of reduced MTT were assayed by measuring the difference in absorbance between 570 and 690 nm. Data are expressed as the percentage reduction in MTT with respect to the control ± SD from five different experiments carried out in triplicate. The MTT reduction values were used for IC50 estimation by Microsoft Office Excel 2013 software.

### 4.4. Cell Cycle Analysis

Using Propidium Iodide (PI) as a well-known nuclear stain, EA.HY926 cells were analyzed according to their DNA content with the purpose of recognizing potential changes in cell cycle progression. Upon completing the treatment, cells were first detached by trypsin and then collected by centrifugation (1.300 RPM—5 min). Subsequently, pellets were washed once in PBS before being permeabilized with 70% ice-cold ethanol/PBS. Samples were finally stored at −20 °C until analysis. An appropriate volume of staining solution containing 15 μg/mL of PI plus 20 μg RNaseA in PBS was used to resuspend cells after the spin cycle (1.300 RPM—5 min). Thereafter, cells were analyzed using FACSCelestaTM (BD Bioscience, San Jose, CA, USA) acquiring at least 10 K events for each single sample. The percentage of cells with a DNA content equal to 2n (G0/G1 phase), 2n–4n (S phase), and 4n (G2/M phase) was defined by gating the respective subpopulations on a histogram plot. A similar procedure was also applied to assess the subG1 phase, namely cells exhibiting a DNA fragmentation (<2n). All biological replicates were carried out in triplicate.

### 4.5. Detection of Intracellular ROS

Intracellular ROS detection was performed using 2′,7′-dichlorofluorescin diacetate (DCFH-DA) assay. Cells cultured in 24-well plates were incubated with DCFH-DA 10 µM for 30 min and lysed with Tris-HCl 0.5 M, pH 7.6, 1% SDS. Controls were performed using untreated cells and cells exposed to 20 mM H_2_O_2_. In oxidative conditions, DCFH-DA was converted to the fluorescent molecule 2′,7′-dichlorofluorescein (DCF). DCF fluorescence intensity was recorded at a 530 nm emission wavelength upon an excitation wavelength of 488 nm using the Perkin Elmer Life Sciences LS 55 spectrofluorometer. Data are expressed as average ± SD from five different experiments carried out in triplicate.

### 4.6. Cellular Nuclear Extraction

Control and treated cells (1 × 10^6^ cells) were harvested by centrifugation, washed with ice-cold PBS, resuspended in lysis buffer (10 mM HEPES pH 7.5, 10 mM KCl, 0.1 mM EDTA, 1 mM dithiothreitol, 0.5% Nonidet-40, and 0.5 mM PMSF, along with the protease and phosphatase inhibitor cocktail) and incubated on ice for 20 min, allowing them to swell. Then, tubes were vortexed, and cells were centrifuged at 12,000× *g* at 4 °C for 10 min. The supernatant, containing the cytosolic fraction, was transferred into a new tube. The pellet nuclei were washed threefold with the cell lysis buffer, resuspended in the nuclear extraction buffer (20 mM HEPES pH 7.5, 400 mM NaCl, 1 mM EDTA, 1 mM DTT, 1 mM PMSF with protease and phosphatase inhibitor cocktail), and incubated for 30 min on ice. After 15 min of centrifugation at 12,000× *g* at 4 °C, the supernatant (the nuclear extract) was recovered. The BioRad assay reagent (Bio-Rad, Hercules, CA, USA) was used to quantify the protein concentration. The nuclear and cytoplasmic extracts obtained were tested for cross-contamination through immunoblotting for HDAC1 and tubulin, respectively.

### 4.7. Immunoblotting

SDS-PAGE (10%) under reducing conditions was used to separate protein cellular extracts (25 μg) that were then transferred to a polyvinylidene difluoride membrane in transfer buffer (25 mM Tris, 192 mM glycine, 20% methanol, 0.1% SDS). The blots were incubated in 5% no-fat dry milk (A0530; AppliChem) blocker for 1 h at room temperature and then incubated overnight at 4 °C with specific primary antibodies. After 1 h of incubation with corresponding horseradish peroxidase-conjugated secondary antibodies, immunocomplexes were revealed using an enhanced chemiluminescence detection kit (Elabscience Biotechnology, Houston, TX, USA) and acquired using Chemi Doc XR (Biorad, Hercules, CA, USA). The relative intensity of protein bands was quantified using a Gel Doc XR System (Biorad, Hercules, CA, USA). Data analysis was performed by comparing each sample with the control, and the normalization was achieved using the housekeeping gene.

### 4.8. Statistical Analysis

Stata software (Version 13.0; StataCorp LP., College Station, TX, USA) was used for statistical analyses. For treatments that were significant from variance analysis (ANOVA), Tukey’s test was performed. Results are represented as the mean ± SD Statistical significance was set at *p* < 0.05.

## 5. Conclusions

Genistein is shown to protect endothelial cells by MG-induced cytotoxicity by the inhibition of intracellular ROS production. Protection from oxidative stress leads to the inhibition of cellular apoptosis via the deactivation of ERK/p38 MAPK pathways. In this respect, our findings identify promising beneficial properties for the possible use of genistein as a therapeutic supplement aimed at preventing the risks of endothelial dysfunction and vascular complications in diabetes.

## Data Availability

The original contributions presented in the study are included in the article; further inquiries can be directed to the corresponding author.
